# Risk of Low Birth Weight According to Household Composition in Brussels and Montreal: Do Income Support Policies Variations Explain the Differences Observed between Both Regions?

**DOI:** 10.3390/ijerph18157936

**Published:** 2021-07-27

**Authors:** Mouctar Sow, Myriam De Spiegelaere, Marie-France Raynault

**Affiliations:** 1 School of Public Health, University of Montreal, Montreal, QC H3N 1X9, Canada; marie-france.raynault@umontreal.ca; 2 School of Public Health, Université Libre de Bruxelles (ULB), 1070 Brussels, Belgium; mdespieg@ulb.ac.be; 3 Lea-Roback Research Centre on Social Inequalities in Health, CRCHUM, Montreal, QC HCX 0C1, Canada

**Keywords:** perinatal inequities, poverty, social policy, income support policies, welfare, health impact assessment, natural experiments, pregnancy outcomes, low birth weight

## Abstract

Variations in social policy between countries provide opportunities to assess the impact of these policies on health inequities. This study compares the risk of low birth weight in Brussels and Montreal, according to household composition, and discusses the impact of income support policies. For each context, we estimated the impact of income support policies on the extent of poverty of welfare recipients, using the model family method. Based on the differences found, we tested hypotheses on the association between low birth weight and household composition, using administrative data from the birth register and social security in each region. The extent of poverty of welfare families differs according to household composition. In Quebec, the combination of low welfare benefits and larger family allowances widens the gap between households with children and those without children. The risk of LBW also differs between these two contexts according to the number of children. Compared to children born into large welfare families, first-born children are more at risk in Montreal than in Brussels. In addition to the usual comparative studies on the topic, our study highlights the importance of an evaluative perspective that considers the combination of different types of income support measures to better identify the most vulnerable households.

## 1. Introduction

### 1.1. Why Study the Impact of Social Policies on Social Inequities in Health?

Social inequities in health (SIH) are observable gaps in health by social status. These gaps are avoidable because they result from the inequitable distribution of resources that enable people to have greater control over their living conditions and health. Reducing them is a necessity for society, health, and moral and economic reasons [1,2,3,4].

While several types of resources contribute to health inequalities, economic resources are central because they can be easily “transformed” into other types of resources [5,6,7,8]. They determine access to material goods and influence social participation. They also influence the health status of the general population, particularly during critical periods such as pregnancy and childhood [9,10,11,12].

Through their impact on households’ economic resources, social policies constitute a major lever for reducing household poverty and economic inequalities through the effects of wealth redistribution [4,5,13,14]. Thus, they constitute a lever for improving the health of populations and reducing health inequities. One major recommendation to reduce these inequities is to improve the health of children as early as possible, particularly the most disadvantaged, through policies that improve family income and help reduce the intergenerational transmission of poverty.

### 1.2. Characteristics of Income Support Policies

Policies that contribute to improving family income have three important characteristics: (a) they combine many measures implemented within the framework of various social policies pursuing different objectives [15,16,17,18,19]; (b) they are implemented according to various criteria of selectivity and generosity, which vary between measures and between countries [15,18,20,21].

These measures include those for non-working and low-income households: unemployment benefits, social assistance, work incentives, and other support (housing/transport/health care). There is also specific support for families with children within the family policy framework, such as family allowances and measures to reconcile work and family life (care policies, maternity and parental leave). These forms of government support are delivered in various ways including direct financial assistance, transfers and tax deductions, and subsidized services (housing, transport, etc.) [18,20,22,23,24]. The combination of these forms varies between countries. A comparative analysis of the influence of income support policies on poverty should consider both dimensions. A comparison of only one component may offer only a partial view of the state’s contribution to family welfare.

Numerous selectivity and generosity criteria guide the selection of families benefiting from one or various measures and the amount of assistance granted. These are mainly income level, occupation status, and family composition (single parenthood and the number and age of children). Other criteria can also be considered, such as the presence or absence of a disability and citizenship or residence status. These targeting generosity criteria differ according to the measure considered and the country, which leads to a differential impact of income support policies on the income of different types of families across countries [15,16,17,19,25]. For example, a single-parent family with two children and no employment income is not helped in the same way (measures for which it is eligible and amount of assistance) depending on whether it resides in Belgium or Quebec. It is important to highlight this effect of targeting measures to better appreciate the impact of income support policies on the situations of different types of households.

### 1.3. Assessing the Impact of Social Policies on SIHs: Limitations of Usual Comparative Studies

Evaluating the impact of social policies on health inequities in different contexts is a complex task, due in particular to the difficulty—impossibility, even—of setting up randomized studies for ethical reasons. Variations in social policies across countries provide opportunities for comparative studies on the issue, based on natural experiments [8].

However, comparative studies focusing on the effect of social policies on health and health inequities analyze the correlation between the characteristics of the social protection system and health indicators. They are most often based on typologies of welfare state regimes, which make it possible to categorize the social protection systems of industrialized countries, and analyze the correlations between these regimes, poverty rate, and health indicators [26,27,28,29]. The classification proposed by Esping-Andersen, which several authors have adapted and adopted, is the most influential typology [21,26,27,30,31].

This classification is based on three principles: the degree of influence of the market on the well-being of individuals, especially the most vulnerable; the influence of state intervention on social stratification, which favors or diminishes inequalities between social classes; and the public-private mix, i.e., the relative contributions of the state, family, community, and the market to individuals’ well-being. The operationalization of these principles has enabled distinguishing three main types of regimes: social-democratic, conservative, and liberal.

These typologies provide an interesting starting point for analyzing the relationship between different social protection systems and the health of populations. However, note that these systems are ideal types, and thus, do not reflect the full reality and diversity of situations. States may differ on one or more social policy dimensions. As noted earlier, the mix of income support policies (components, targeting, generosity) differs between countries. Income support measures may also differ within countries. Quebec, for example, has a family policy that stands out from many Canadian provinces [32,33].

Studies based on welfare state regimes do not reveal these differences between countries. These global, macro-social analyses do not focus on the specificities of the measures. In other words, these typologies do not reveal the targeting effect of income support policies. As a result, they do not contribute to a better understanding of how the articulation of income support policies influences household poverty and contributes to SIHs [26,27,28,29]. Furthermore, these studies often analyze the effect of welfare state regimes on the health of the general population and not the effects on health inequalities [26].

Since they cover the entire range of state action, these studies do not directly help identify concrete courses of action that can reduce poverty and SIHs in different contexts. The institutional approach [12], which analyzes the impact of specific policies on the targeted socio-demographic groups [12,14], makes it possible to overcome this limitation.

### 1.4. Objectives

This study, which is a part of a larger research project [5], particularly concerns itself with welfare households. The analysis aims to understand how income support measures in Belgium and Quebec make certain types of households on social assistance more or less vulnerable to poverty and how they contribute to perinatal inequities. The comparison of income support policies in force between Belgium (country) and Quebec, as a distinct entity of Canada, is justified by the specificities of the province, which has a great deal of autonomy in the design of social policies, implying notable differences with the rest of Canada at several levels [32,33,34].

More specifically, this study answers three main questions:

(1) How do income support policies in Belgium and Quebec influence the disposable income of welfare families, according to household composition?
(2) Does the risk of LBW among mothers on welfare vary according to the household’s composition in Brussels and Montreal?
(3) To what extent can the similarities and differences observed between the two regions be explained by the conception of income support measures?

The perinatal indicator considered here is low birth weight (i.e., weight < 2500 g), which is associated with infant mortality and influences a child’s development and their health condition well into adulthood [35]. Low birth weight (LBW) is usually used to study the impact of socioeconomic status or income support measures on perinatal inequalities in different contexts [36,37].

## 2. Description of Income Support Measures in Belgium and Quebec

The income of social assistance recipients in Belgium and Quebec stems mainly from last-resort financial assistance and family allowances. The benefits granted for each of these measures vary according to household composition.

### 2.1. Last-Resort Financial Assistance

In Quebec, social assistance recipients are distinguished primarily by their marital status. The amount allocated to couples is higher than that allocated to single people. The basic benefit does not consider the presence or absence of children, but a supplement for temporary work constraints is granted to single-parent families with a child aged less than five years [38].

In Belgium, the amounts of the social assistance (‘Revenu d’intégration sociale’: RIS) also vary according to the marital situation and consider the presence of dependent children. Single persons (isolated category), couples without children (cohabitants category), and households with at least one dependent child are distinguished, regardless of marital status (head of household category). A couple with two members receiving social assistance (two cohabitants’ benefits) and a household with at least one child receive almost the same amount. Therefore, two levels of benefits can be distinguished: one amount for a single person and one for all other family configurations [39,40].

### 2.2. Family Allowances

In Quebec, family benefits come from both the federal and provincial governments. They are highly progressive depending on household income. In 2016, federal benefits were significantly increased. Households on social assistance receive the maximum amount at each government level. Supplements are provided for single-parent families [41,42].

In Belgium, until 2019, family allowances were characterized by a universal basic benefit, to which were added various types of supplements for families in certain categories (social assistance recipients, unemployed, convalescents, single-parent families, etc.) [43,44]. Recently, the system of family allowances has been decentralized and therefore currently differs between the three regions of the country. In all three regions, the basic benefits have been increased, the increase in the amounts according to the rank of a child has been abolished, and a greater progressivity according to household income has been introduced (instead of the social categories previously considered). Households on social assistance are entitled to a social supplement, and in the Brussels region, single-parent families with several children receive a supplement [44].

## 3. Materials and Methods

Two case studies were conducted and analyzed from a comparative perspective. The analysis was carried out in two stages. First, we assessed the impact of income support policies on the standard of living of different types of welfare households in each context. Then, based on the differences observed, we developed and tested hypotheses on the association between LBW and household composition in Brussels and Montreal, using a combination of administrative data provided by birth registers as well as socioeconomic and demographic data from both regions.

### 3.1. Impact of Income Support Measures on the Standard of Living of Welfare Households

#### 3.1.1. Model Family Method

The analysis is based on the model family method. This method consists of calculating and comparing the disposable income of different types of households considering all measures from which these households benefit in each context. In this way, it is possible to identify households for which a state is doing better or worse. It is also possible to show the relative impact of different types of measures in each state. The method is based on typical cases and is therefore illustrative [15,17,20,33,45].

We compared the disposable income of households with no work income that receive welfare in Belgium and Quebec. Eight types of households were considered: single people, couples without children, and single-parent and two-parent families with one, two, or three children under the age of 6. The calculation of the disposable income takes account of the monetary measures benefiting these households in both states: welfare, tax credits for welfare recipients, and family allowances.

The disposable income of welfare families in Belgium and Quebec were converted at purchasing power parity (PPP) in order to make them comparable. PPP equalizes the purchasing power of different currencies by eliminating differences in price levels between countries [46]. Thus, purchasing power parity allows us to directly compare the standard of living of Belgian and Quebec welfare households.

However, this comparison does not inform us on the living standard inequalities between these households and the general population for each context. Such information is important because the impact of low income on health and health inequities is explained not only by low purchasing power, i.e., the difficulty of acquiring goods and services, but also by the psychosocial impact of income inequalities and social comparison [6,47]. The relative gap between the disposable income of welfare households and the poverty threshold (poverty gap) allows us to approximate how far the income of welfare households is from that of the general population [48]. Following this logic, we compared the poverty gap of different types of welfare households in Belgium and Quebec.

#### 3.1.2. Disposable Income and Poverty Gap Estimates

In Belgium, the disposable income of welfare families includes the amount of social assistance (‘Revenu d’intégration sociale’: RIS) [40], guaranteed family allowances [44], and the dependent child tax credit [49].

In Quebec, disposable income includes amounts from the Quebec social assistance program, provincial and federal family benefits, and refundable tax credits. Single-parent families are entitled to an additional amount of social assistance for temporary work constraints [50,51]. In 2015, federal family benefits included the Universal Child Care Benefit (UCCB) [52] and the Canada Child Tax Benefit (CCTB) [42]. At the provincial level, the calculations refer to the Child Support Program (“Soutien au enfants”) [41]. Two types of credits available to low-income households were considered, namely the credit for the goods and services tax (GST) [53] and the solidarity tax credit [54]. For family benefits and tax credits, the benefits are calculated based on the tax return for the period from 1 July to 30 June the following year. Thus, the amount received by households over the same year differs between the first and last six months of the year.

### 3.2. Epidemiological Analyses: Associations between LBW and Household Composition

The analysis conducted in the previous step highlighted the differences in purchasing power of welfare households in Belgium and Quebec according to the number of children and the number of parents. Based on the differences found, we developed hypotheses on the association between LBW and household composition in Brussels and Montreal among welfare families. We tested these hypotheses using administrative data in each region. The analyses carried out made it possible to describe the socio-demographic profile of families on social assistance in Brussels and Montreal and to study the association between LBW and household composition among these families.

#### 3.2.1. Data Sources and Study Population

We combined administrative databases from different sources for both regions. The database used for Brussels combines information from the birth records, the national migration register, and the social security system. Information on welfare recipients is recorded on a quarterly basis. It was therefore possible to identify the parents who received welfare during the quarter in which the child was born. The data cover a period spanning from 2005 to 2010. During this period there were 97,844 singleton live births in Brussels, 6831 of which were performed by mothers who were receiving welfare at the time of delivery. For Montreal, two databases were used. The first covers birth records from 2003 to 2012, which amounted to 214,620 single live births. The second database concerns births among families receiving welfare benefits, specifically those where mothers were welfare recipients in the month of delivery. It is the result of a combination of data from the Ministry of Labour, Employment, and Social Solidarity and the Birth and Death Register. The data cover a period spanning from 2010 to 2016. During this period, there were 10,298 single live births among mothers receiving welfare. These data combinations are, to our knowledge, the first of their kind in Belgium and Quebec.

#### 3.2.2. Variables and Statistical Analysis

Low birth weight (LBW) was analyzed according to the number of children and the mother’s marital status in Brussels and Montreal. The relationship status separates children whose mother is a single parent and those whose mother lives with their partner. Parity, which refers to the number of previous live births, was used as a proxy for the number of children in the household. We compared the risk of LBW among first-born children with that of the subsequent children. Epidemiological studies on the link between parity and perinatal health show that the prevalence of LBW is higher in primiparous mothers (first-born), then decreases in multiparous mothers, and increases again in large multiparous mothers [55]. The comparison of the results in the two regions pays close attention to this pattern.

In each region, logistic models were developed to analyze the association between LBW and (1) number of children and (2) the relationship status of the mother. Other variables were considered to describe births and adjust the multivariate analyses: the mother’s birthplace, the mother’s age at the birth of child, and maternal education, as well as the child’s sex. The patterns observed in welfare households were compared with those in the general population. Among welfare households, the analysis also takes the influence of time on welfare into account. This variable draws a distinction between mothers who have been welfare recipients for at least two consecutive years and those who were for a shorter period. First, the results were adjusted for time on welfare. Then, stratified analyses, according to time on welfare, were performed in order to better appreciate the risk of low birth weight among long-term welfare recipient mothers.

## 4. Results

### 4.1. Characteristics of Income Support Measures in Belgium and Quebec and Impacts on the Standard of Living of Welfare Households

Income support policies differ significantly between Belgium and Quebec, namely in terms of the generosity of income support measures. Table 1 shows, on the one hand, the generosity of income support in Belgium and, on the other hand, the importance of family allowances in Quebec.

#### 4.1.1. Comparison of Household Purchasing Power by Number of Children and Relationship Status

When combining these two types of policies in the two contexts, the main difference observed pertains to households without children. The latter receive much lower benefits in Quebec than in Belgium. As far as households with children are concerned, their purchasing power is similar in both states, particularly among families with several children. In a way, for Quebec families receiving welfare, the larger family allowances compensate for the lower welfare benefits and bridge the gap with Belgian households. They do, however, widen the gap between households with children and those without children.

While the purchasing power of households differs between Belgium and Quebec according to the presence or absence of children, patterns according to relationship status are similar in the two states. In Quebec, however, the purchasing power of two-parent families is slightly higher than that of single-parent families with the same number of children. In Belgium, the figures for these two types of households are similar. This can be explained by the fact that, in Belgium, the generosity of welfare benefits and tax credits granted to households with children does not differ according to the parent’s relationship status. In other words, they do not take the presence of a second adult into account, which puts two-parent families at a disadvantage [27].

#### 4.1.2. How Does the Standard of Living of Welfare Households Compare to That of Other Households in Belgium and Quebec?

The relative gap between the disposable income of welfare families and the poverty threshold (poverty gap) allows us to approximate how far the income of welfare families is from that of the general population according to household composition [49].

The main results (Table 2) show that the disposable income of welfare households does not reach the poverty threshold in both contexts, but that the poverty gaps are more pronounced in Quebec than in Belgium. As was seen in the case of purchasing power, the number of children greatly influences the poverty gaps in Quebec. Case in point: the absolute difference in the poverty gap between a two-parent family with two children and a couple without children reaches 20 percentage points in Quebec, whereas in Belgium, the difference amounts to 2 percentage points. In the Belgian context, the poverty gap is more sensitive to the couple’s relationship status. Among households with children, the disposable income of single-parent families is closer to the poverty threshold than that of two-parent families.

### 4.2. Epidemiological Analyses: Associations between LBW and Household Composition

#### 4.2.1. Working Hypotheses

The previous analysis demonstrates that the combinations of income support measures further disadvantage certain types of welfare households in each state. In Quebec, childless households are particularly vulnerable to poverty, both in terms of purchasing power and poverty gap, while in Belgium income support policies put two-parent families at a disadvantage. Children born in these two types of households may be more vulnerable to LBW compared to other household types. It has indeed been well established that poverty before and during pregnancy increases the risk of unfavourable pregnancy outcomes (low birth weight, preterm birth, stunted growth) [22,31].

Table 3 and Table 4 compare the socio-demographic characteristics of newborn children among general population and welfare recipients in both regions. Table 5 compare the risk of LBW according to the number of children and mothers’ marital status among welfare recipients.

#### 4.2.2. Characteristics of Births in Montreal and Brussels

Brussels mothers are more likely to be single, poorly educated, immigrants, and to have more than two children, while in Montreal, there is a greater number of primiparous mothers as well as mothers under 20 or over 39 years of age (Table 3).

Beyond this general pattern, we observed significant differences between households in both regions according to the mothers’ marital status. In both regions, single-parent families are more vulnerable than two-parent families, in terms of primiparity, the young age of the mother, and her education level. For these last two characteristics, the differences between both types of households are higher in Montreal than in Brussels. For example, with regard to the level of education of Montreal mothers, 53% of single mothers and 22% of mothers in a relationship did not pursue an education beyond Secondary V (Secondary V corresponds to 11 years of study). The corresponding figures in Brussels are 33% and 29% respectively. In addition, the proportion of very young mothers among single mothers in Montreal is quite high. The proportion of immigrant mothers among single parents and couples, however, is similar for each region (Table 3).

The characteristics of welfare households (Table 4) differ from those of the general population in certain respects. Single parents and immigrant mothers are overrepresented among welfare households in both regions. In contrast to the general population, the proportion of large families is higher among welfare households in Montreal.

The differences we observed among the general population were also found among welfare households: there are a higher proportion of single-parent families in Brussels and a higher proportion of very young mothers among single parents in Montreal. The differences between single-parent and two-parent families, which are more significant in Montreal than in Brussels, are even more pronounced among welfare households. For example, 12% of single mothers receiving welfare in Montreal are under 20 years of age, compared with 1% of two-parent families. The corresponding figures for Brussels are 8% and 7%, respectively.

Comparing the education level of welfare mothers between both regions was quite difficult, since a fairly large portion of values was missing for Montreal. The pattern that emerges, however, is that single mothers in Montreal are more vulnerable. Even if we were to include the missing values in the denominator, the proportion of poorly educated single mothers would still be higher in Montreal than in Brussels (Table 4). The proportion of mothers who have been on welfare for at least two consecutive years is higher in Brussels than in Montreal. In Brussels, half of the single mothers are in this situation.

#### 4.2.3. Associations between LBW and Household Composition among Welfare Families

The association between LBW and household composition shows some similarities, but also some important differences (Table 5). Being first-born is associated with a high prevalence of LBW in Brussels and Montreal. However, we observed one notable difference between these two regions: compared to first-born children, children of birth rank 3 and above do not show a significant reduction in LBW risk in Brussels, whereas in Montreal, they seem to be significantly protected from this pregnancy outcome. The extent of this protection increases after adjusting for the mother’s education level and birthplace.

Single parenthood is associated with a higher risk of LBW in both regions. The excess risk associated with single parenthood is higher in Montreal than in Brussels. This difference is slighter after adjusting for the mother’s education level and birthplace (Table 5).

#### 4.2.4. The Influence of Time on Welfare: A Marked Impact among Primiparous Montreal Women

Table 5 shows that in Montreal, the association between LBW and birth rank among welfare recipients varies after adjusting for maternal age and time on welfare, with the excess risk associated with primiparity increasing significantly. To better visualize this variation, let us take births of rank 3 and 3 + as a reference category. We then see that the crude odds ratio of first-born babies versus babies born into large families is 1.50 (1.22–1.85). It then rises to 1.84 (1.44–2.34) after adjusting for the mother’ age and time on welfare. In Brussels, the odds ratio of LBW does not change significantly after adjusting for these two factors, as it goes from 1.14 (0.90–1.45) to 1.25 (0.97–1.60). These differences between the two regions suggest that first-time mothers who have been on welfare for a relatively long time (at least two years) and younger mothers are more vulnerable in Montreal than in Brussels, compared with multiparous mothers in both regions. This would explain the significant excess risk associated with primiparity in Montreal.

Table 6 provides a better appreciation of the influence of time on welfare on the risk of LBW among primiparous and multiparous mothers in both regions. We can see that long-term welfare dependence particularly weakens Montreal primiparous mothers, with the prevalence of LBW increasing from 5.87% to 9.74%. In Brussels, the prevalence of LBW increases from 7.26% to 7.80% depending on the time on welfare. Among the first children born to mothers who received welfare, the prevalence of LBW is also higher in Montreal than in Brussels (9.74% vs. 7.80%), unlike all other groups of children.

Among multiparous mothers (2nd and subsequent births), we noticed the opposite pattern to that observed among primiparous mothers. Indeed, a mother’s long-term dependence on welfare is accompanied by a higher marked increase in prevalence of LBW in Brussels (4.79% vs. 6.63%) than in Montreal (4.20% vs. 4.55%).

## 5. Discussion

On the epidemiological level, two main findings emerged from this study. The first is that there is a greater risk of LBW of the first-born children in Montreal than in Brussels, compared to the following births. The second concerns the differences observed between the regions before and after the adjustment for the excess risk of LBW associated with single parenthood. The discussion addresses these two aspects.

### 5.1. Marked Vulnerability of First-Born Children in Montreal

Epidemiological studies show that the prevalence of adverse pregnancy outcomes is higher in primiparous mothers (first-born), then decreases for subsequent births, and increases again in large multiparous mothers [26]. In general, our results confirm this trend in both regions. However, in Montreal, among welfare families, the increase of the prevalence remains slight, while in Brussels the increase is relatively large. The differences between large families in the two regions are even more pronounced when comparing households that have been long-term welfare recipients. For Montreal mothers, there is an opposite pattern to the usual findings, as the prevalence of LBW declines among births of rank 3 and 3 + compared with those of rank 2 (4.92% vs. 4.32%). In Brussels, the pattern of increase applies (6.04% vs. 7.18%).

**Figure 1:**
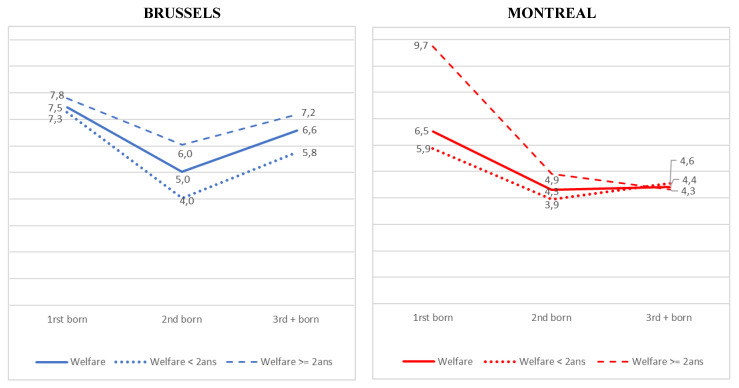
Prevalence of LBW according to birth order in Brussels and Montreal.

All in all, the greater excess risk of LBW among first-born babies in Montreal than in Brussels can be explained, on the one hand, by the contrasting situation between large families in the two regions, and on the other hand, by the impact of long-term welfare dependence, which amplifies the differences between large families and makes primiparous Montreal mothers more vulnerable. Figure 1 illustrates these differences.

These results are consistent with those of the comparison of household poverty, which show a significant vulnerability of childless households in Quebec due to the low generosity of welfare, while the generosity of family allowances largely improves the living standard of households with children. Furthermore, the analysis reveals the importance of taking the duration of poverty into account in order to better evaluate the impact of income support measures on LBW inequalities.

### 5.2. Differences in Risk According to Household Status: The Role of Compositional Actors

The adjustment for the mother’s education level and birthplace greatly influenced the differences between both regions with respect to the risk related to single parenthood (Table 5). In Montreal, this risk decreased considerably when adjusting for these two factors. In Brussels, the adjustment for the mother’s education level and birthplace did not alter the figures observed.

The excess risk associated with single parenthood in Montreal can be explained, among other things, by the higher proportion of native-born mothers in this group (61%), whereas only 13% of mothers living with their partner are born in Canada (Table 2). Several studies have shown that, for the most disadvantaged households, the risk of LBW is greater among native-born mothers than among immigrant women [32,33,34]. In a similar vein, a link can be made between the relatively high proportion of poorly educated mothers among Montreal single-parent families compared to two-parent families (53% vs. 22%). Stratified analyses according to the mother’s birthplace and education level could help us better understand the influence of these factors on the risk associated with single parenthood in both regions.

While the mother’s education level and birthplace largely explain the differences between both regions in terms of risk of LBW associated with single parenthood, our results show that income support policies play a role in this too. Among welfare households, the additional risk associated with single parenthood is lower in Brussels than in Montreal. To a large extent, this difference can be explained by differences between couples in the two regions: they appear to be relatively more vulnerable to LBW in Brussels than in Montreal. The differences of prevalence between both regions, Brussels being at a disadvantage, are more marked among couples than among single-parent families (Table 5). The relatively high vulnerability of couples in Brussels to LBW is consistent with the results on poverty, which show that two-parent families in Brussels experience a higher intensity of poverty. Moreover, the poverty gaps among two-parent families and single-parent families vary according to the number of children. Distinguishing the risk of LBW associated with single parenthood according to the number of children could help better appreciate the impact of income support measures.

### 5.3. Contribution to the Evaluation of the Impact of Social Policies on SIHs

We compared contexts with important differences in the design of income support policies. This comparative approach and the use of the model family method yielded important insights into the characteristics of income support policies and their impacts on SSI at birth in the two contexts. Compared to usual evaluative studies, the main contribution relates to the fact that the analysis takes into account the combination between two types of income support policy and highlights their relative impact on income inequalities between different types of households in Belgium and Quebec.

Important lessons can be drawn. First, the findings of studies that focus on comparing a specific type of policy deserve to be qualified. Indeed, several types of measures with different objectives and strategies are found to contribute in a complementary way to the objective of supporting household income and improving living conditions. The effects of social assistance policy, which aims to ensure a minimum income, and those of family allowances, which are intended to reduce the cost of children, combine to protect poor households with children. A comparison of allowances alone would have concluded that children in households receiving welfare are better protected in the Quebec context. Conversely, comparisons that focus solely on minimum income protection measures [28,29] would overstate the differences between households in the two contexts in favor of Belgium.

The second lesson, which follows directly from the first, concerns the importance of distinguishing between the situations of families according to the number of children and the couple situation. For example, the extent of poverty among single-parent families differs according to the number of children. Studies that compare the poverty and health indicators of single-parent families to those of two-parent families do not reveal these differences [30,31]. Our study also shows that comparing inequalities by birth order in different contexts may reflect differences in the generosity of income support between households with and without children.

In short, in addition to studies that take a global approach to the impact of all government action [22,23] and those that consider the impact of only one type of policy [28,30], our research highlights the importance of a third evaluative perspective that considers the articulation of income support measures to better identify the poorest and most vulnerable households with respect to perinatal health in different contexts.

Another noteworthy element is the linkage of administrative data, in this case health and social security data, in both regions. As these data reflect the surveillance systems regarding these issues in the two regions, the approach has enriched our understanding of the contexts. It has also made it possible to have unpublished databases that cross-reference different types of information on beneficiaries in Brussels and Montreal.

### 5.4. Limits

The comparison of the disposable income of households receiving social assistance does not consider indirect services (non-monetary services) such as social housing and assistance with energy, heating, or transportation. Simulations that include such services would increase the understanding of the impact of monetary measures in different contexts on the disposable income of welfare households.

Regarding the comparison of health inequalities, the unavailability of certain information in our databases did not allow us to explore certain hypotheses further. For example, information on smoking habits or alcohol consumption during pregnancy would have enabled estimating the extent to which these contribute to the differences observed in health inequalities at birth between the two contexts.

Furthermore, the analysis carried out concerns LBW. It would be relevant to extend this type of analysis to other indicators of adverse pregnancy outcomes, such as prematurity and SGA.

## 6. Conclusions

The use of the model family method made it possible to highlight income inequalities between welfare households in Belgium and Quebec, compare the relative impact of different income support measures in both contexts, and to develop hypotheses on the association between LBW and household composition.

The main difference concerning the extent of poverty pertains to households without children. In Quebec, the combination of low welfare benefits and larger family allowances widens the gap between households with children and those without children. The risk of LBW also differs between these two contexts according to number of children. Compared to children born into large welfare families, first-born children are more at risk in Montreal than in Brussels. Long-term reliance on welfare further weakens primiparous mothers in Montreal. There are also significant differences between the two regions associated with single parenthood, both in terms of household characteristics and risk of LBW.

Our analysis shows the relevance of taking the specific combination of different income support measures into account to better identify the most or least vulnerable households between different contexts. Such an approach allows us to better assess the impact of income support policies on health inequalities at birth. This study is the first, to our knowledge, to use such an approach. 

## Figures and Tables

**Table 1:** Welfare and family allowance benefits (PPP 2015) in Belgium vs. Quebec.

	Single-Parent Households			Two-Parent Households			Childless Households	
	1 Child	2 Children	3 Children	1 Child	2 Children	3 Children	1 Adult	2 Adults
**Welfare**								
Belgium	16,456	16,456	16,456	16,456	16,456	16,456	12,342	16,456
Quebec	7183	7183	7183	9183	9183	9183	5923	9183
**Family allowances**								
Belgium	2113	5115	9269	2113	5115	9000	0	0
Quebec	7079	12,335	17,595	6414	11,670	16,930	0	0
**Tax Credit**								
Belgium	469	1075	1613	469	1075	1613	0	0
Quebec	1404	1611	1817	1612	1819	2026	1094	1405
**TOTAL**								
**Belgium**	**19,038**	**22,646**	**27,338**	**19,038**	**22,646**	**27,069**	**12,342**	**16,456**
**Quebec**	**15,666**	**21,128**	**26,595**	**17,209**	**22,671**	**28,139**	**7017**	**10,588**

PPP conversion rates (OCDE 2015): Belgium = 0.800 and Canada = 1248.

**Table 2:** Poverty gap among welfare households in Belgium and Quebec in 2015.

	Single-Parent Households			Two-Parent Households			Childless Households	
	1 Child	2 Children	3 Children	1 Child	2 Children	3 Children	1 Adult	2 Adults
**Belgium**								
Poverty threshold *	21,738	26,754	31,770	30,098	35,115	40,131	16,721	25,082
Poverty gap	**−12%**	**−15%**	**−14%**	**−37%**	**−36%**	**−33%**	**−26%**	**−34%**
**Quebec**								
Poverty threshold *	24,586	30,259	35,933	34,042	39,715	45,389	18,912	28,368
Poverty gap	**−36%**	**−30%**	**−26%**	**−49%**	**−43%**	**−38%**	**−63%**	**−63%**

* Poverty threshold in each context converted to purchasing power parity (PPP).

**Table 3:** Socio-demographic characteristics of mothers in the general population.

	BRUSSELS (2005–2010)			MONTREAL (2003–2012)		
	COUPLE	SINGLE	TOTAL	COUPLE	SINGLE	TOTAL
**N**	74,351	14,326	88,677	187,533	20,696	208,249
% of births	83.84	16.16	100	90.06	9.94	100
**Parity (*n*)**	73,876	14,254	88,130	187,553	20,696	208,249
1st born (%)	45.24	51.92	46.32	48.07	53.14	48.57
2nd born (%)	31.41	25.76	30.49	34.10	26.50	33.34
3rd + born (%)	23.36	22.32	23.19	17.83	20.36	18.08
**Maternal age (*n*)**	74,351	14,326	88,677	187,533	20,696	208,249
<20 (%)	1.33	2.30	1.59	1.36	9.57	2.18
≥40 (%)	4.10	3.88	4.55	6.63	6.34	6.60
**Maternal birthplace (*n*)**	73,495	14,123	87,618	183,723	20,227	208,249
Born in Belgium or Canada (%)	42.50	43.38	42.64	46.51	47.88	46.44
**Maternal education (*n*)**	73,874	14,190	88,064	177,066	19,022	196,088
Secondary V max	29.44	33.05	30.03	21.74	53.09	24.78

**Table 4:** Socio-demographic characteristics of mothers on welfare.

	BRUSSELS (2005–2010)			MONTREAL (2010–2016)		
	COUPLE	SINGLE	TOTAL	COUPLE	SINGLE	TOTAL
**N**	2011	3673	5684	5152	5146	10,298
% of births	35.38	64.62	100.00	50.02	49.98	100.00
**Parity (*n*)**	2001	3648	5649	5152	5146	10,298
1st born (%)	53.07	51.26	51.90	23.02	48.31	35.66
2nd born (%)	25.24	24.07	24.48	36.84	25.96	31.40
3rd + born (%)	21.69	24.67	23.61	40.14	25.73	32.94
**Maternal age (*n*)**	2011	3673	5684	5152	5146	10,298
<20 (%)	6.81	8.30	7.78	1.26	11.89	6.57
≥40 (%)	2.49	4.74	3.94	7.36	3.65	5.51
**Maternal birthplace (*n*)**	1993	3634	5627	5152	5146	10,298
Born in Belgium or Canada (%)	20.47	19.18	19.64	12.73	61.54	37.12
**Consecutive time on welfare (*n*)**	2011	3673	5684	5152	5146	10,298
2+ years	32.92	50.31	43.57	33.42	38.57	36.00
**Maternal education (*n*)**	2011	3673	5684	5152	5146	10,298
>Secondary V	45.35	45.36	45.35	20.81	5.71	13.26
Secondary V max	46.64	43.94	44.90	23.91	50.47	37.18
Unknown	8.01	10.70	9.75	55.28	43.82	49.55

**Table 5:** Risk of LBW according to household composition among welfare recipients.

	BRUSSELS				
	% LBW	Crude OR	Adjusted OR ^a^	Adjusted OR ^b^	Adjusted OR ^c^
**Birth rank**					
1st born	7.45	1	1	1	1
2nd born	5.02	0.66 (0.51–0.84) **	0.63 (0.48–0.81) ***	0.65 (0.49–0.84) **	0.62 (0.48–0.81) ***
3rd + born	6.58	0.87 (0.63–1.06)	0.80 (0.62–1.04)	0.81 (0.62–1.05)	0.74 (0.56–0.97) *
**Marital status**	6.63				
Couple	5.45	1	1	1	1
Single	7.28	1.36 (1.08–1.71) **	1.34 (1.06–1.69) *	1.36 (1.07–1.73) *	1.35 (1.06–1.72) *
	**MONTREAL**				
	**% LBW**	**Crude OR**	**Adjusted OR ^a^**	**Adjusted OR ^b^**	**Adjusted OR ^c^**
**Marital status**					
1st born	6.51	1	1	1	1
2nd born	4.30	0.64 (0.52–0.79) ***	0.57 (0.46–0.72) ***	0.61 (0.48–0.76) ***	0.64 (0.48–0.76) ***
3rd + born	4.42	0.66 (0.54–0.82) ***	0.54 (0.42–0.69) ***	0.55 (0.43–0.70) ***	0.58 (0.43–0.70) ***
**Relationship status**	5.13				
Couple	3.80	1	1	1	1
Single	6.45	1.74 (1.45–2.09) ***	1.86 (1.54–2.25) ***	1.45 (1.17–1.80) **	1.43 (1.38–1.57) ***

* ≤ 0.05; ** ≤ 0.01; *** ≤ 0.001. ^a^: adjusted for the sex of the child and the mother’s age at childbirth. ^b^: adjusted for the sex of the child, mother’s age, maternal education, and maternal birthplace. ^c^: adjusted for the sex of the child, age, mother’s age, maternal education, maternal birthplace, marital status, and birth rank.

**Table 6:** Association between LBW and mother’s time on welfare.

	BRUSSELS					
	All Births		1st Born		2nd + Born	
	% LBW	OR (CI 95%)	% LBW	OR (CI 95%)	% LBW	OR (CI 95%)
**Time on welfare**						
<2 years	6.28	1	7.26	1	4.79	1
+2 years	7.13	1.14 (0.94–1.39)	7.80	1.08 (0.83–1.39)	6.63	1.41 (1.03–1.92) *
	**MONTREAL**					
	**All births**		**1st born**		**2nd + born**	
	**% LBW**	**OR (CI 95%)**	**% LBW**	**OR (CI 95%)**	**% LBW**	**OR (CI 95%)**
**Time on welfare**						
<2 years	4.98	1	5.87	1	4.20	1
+2 years	5.40	1.08 (0.90–1.30)	9.74	1.73 (1.28–2.35) ***	4.55	1.08 (0.86–1.37)

* ≤0.05; *** ≤0.001.

## Data Availability

Belgian data are available from the authors upon reasonable request and with permission of Commission for the Protection of Privacy (CPP). Canadian data are available at the Québec Inter-University Center for Social Statistics (QICSS).
